# Estimated pulmonary capillary wedge pressure assessed by speckle tracking echocardiography predicts successful ablation in paroxysmal atrial fibrillation

**DOI:** 10.1186/s12947-016-0049-4

**Published:** 2016-01-27

**Authors:** Masanori Kawasaki, Ryuhei Tanaka, Taiji Miyake, Reiko Matsuoka, Mayumi Kaneda, Shingo Minatoguchi, Takeshi Hirose, Koji Ono, Maki Nagaya, Hidemaro Sato, Yoshiaki Kawase, Shinji Tomita, Kunihiko Tsuchiya, Hitoshi Matsuo, Toshiyuki Noda, Shinya Minatoguchi

**Affiliations:** 1 Department of Cardiology, Gifu University Graduate School of Medicine, 1-1 Yanagido, Gifu, 501-1194 Japan; 2 Department of Cardiology, Gifu Prefectural General Medical Center, Gifu, Japan; 3 Department of Cardiology, Gifu Heart Center, Gifu, Japan; 4 Department of Internal Medicine, Sawada Hospital, Gifu, Japan

**Keywords:** Atrial fibrillation ablation, Recurrence, Pulmonary capillary wedge pressure, Speckle tracking echocardiography

## Abstract

**Background:**

Atrial fibrillation (AF) is associated with left atrial (LA) remodeling caused by pressure and/or volume (LAV) overload. Increased pulmonary capillary wedge pressure (PCWP) represents LA pressure overload. We recently reported that pulmonary capillary wedge pressure (ePCWP) can be estimated by the kinetics-tracking (KT) index that combines LA function and volume using speckle tracking echocardiography (STE), and has a strong correlation with PCWP measured by right heart catheterization (*r* = 0.92). Therefore, we hypothesized that ePCWP is the best echocardiographic predictor of successful AF ablation.

**Methods:**

We enrolled 137 patients with paroxysmal AF (age: 61 ± 10 years) who underwent pulmonary vein isolation. We measured LAV index, LA emptying function (EF) and LA stiffness during sinus rhythm before ablation using STE. PCWP was noninvasively estimated by STE as we previously reported. Parameters were compared between a group with AF recurrence (*n* = 30, age: 59 ± 11 years) and a group with successful ablation (sinus rhythm maintained for >1 year) (*n* = 107, age 61 ± 11 years).

**Results:**

The ePCWP was correlated with PCWP measured by right heart catheterization (*r* = 0.76, *p* < 0.01). Compared with the non-recurrence group (*n* = 107, age: 61 ± 11), the AF recurrence group had significantly increased ePCWP (10.6 ± 3.5 vs 14.6 ± 2.9 mmHg, *p* < 0.01), minimum LAV index (29 ± 12 ml/m^2^ vs 37 ± 14 ml/m^2^, *p* < 0.01) and LA stiffness (0.47 ± 0.33 vs 0.83 ± 0.59, *p* < 0.01), but lower total LA EF (44 ± 11 % vs 39 ± 13 %, *p* < 0.01) before ablation. In multivariate logistic regression analysis, ePCWP was the most significant independent predictor of successful ablation. Using 13 mmHg of PCWP as the optimal cutoff value, the sensitivity and specificity for successful ablation were 73 and 77 % (area under the curve = 0.81), respectively.

**Conclusion:**

The ePCWP that is measured by the combination of LA function and volume before ablation was a better predictor of the successful ablation compared with LA function and volume separately. The ePCWP estimated by STE is useful to predict the successful ablation in paroxysmal AF, and could be useful to improve candidate selection for AF ablation.

## Background

Atrial fibrillation (AF) is the most common sustained arrhythmia and is associated with left atrial (LA) enlargement, remodeling and fibrosis caused by LA pressure and/or volume (LAV) overload [1–3]. AF ablation is an important therapeutic modality because AF ablation has become an effective treatment strategy for patients with drug-refractory AF and is more effective than anti-arrhythmic medications [4, 5].

Many factors such as type of AF, LA size, hypertension, diabetes mellitus, renal function, age, CHADS2, R2CHADS2 or CHA2DS2-VASc score have been proposed as predictors of outcome after AF ablation [6–8]. Recently, left atrial appendage wall-motion velocity and sinus rhythm restoration and arrhythmia noninducibility have been proposed as predictors of outcome after AF ablation [9, 10]. Although echocardiographic parameters have not been fully examined as predictors of ablation outcome except for LA dimension, volume and function [10–15], the ability of LA pressure to predict the success AF ablation is unknown.

We recently reported that estimated pulmonary capillary wedge pressure (ePCWP) has a strong correlation with PCWP measured by right heart catheterization (*r* = 0.92) [16, 17]. ePCWP is estimated by the kinetics-tracking (KT) index that is obtained by the combination of LA function and volume using speckle tracking echocardiography (STE). Therefore, we assessed the hypothesis that ePCWP as well as LA size may be useful predictors for successful AF ablation in patients with paroxysmal AF.

## Methods

### Subjects and study protocol

We enrolled 150 consecutive patients with paroxysmal AF undergoing pulmonary vein isolation (PVI) from June 2012 until January 2014. Paroxysmal AF was defined as self-terminating within 7 days after onset documented by routine electrocardiograms (ECG) or Holter ECG. Patients who met any of following criteria were excluded: an inadequate echo image to analyze LA function and volume, AF when echocardiography was performed, severe mitral regurgitation, or moderate or severe mitral stenosis. Patients who needed evaluation for heart failure underwent right heart catheterization including PCWP measurement. We validated the correlation between ePCWP and PCWP in these patients during sinus rhythm. All patients who underwent PVI were followed at the out-patient clinic of our hospital every month for at least 12 months after the procedure: an ECG was obtained every month and additional Holter recordings were obtained when the patient’s symptoms suggested of AF. Recurrence of AF was defined as any episode of AF lasting >30 s after AF ablation and was documented by ECG or Holter ECG. Transthoracic echocardiography was performed in the left lateral decubitus position by two experienced sonographers just before PVI (within two hours). Right heart catheterization was performed in the supine position without sedatives. The present study was approved by the institutional review board of Gifu Heart Center and all patients gave written informed consent before participation. The present study conforms to the principles outlined in the Declaration of Helsinki.

### Radiofrequency catheter ablation

AF ablation was performed using an approach well documented by Eitel et al. [18]. Patients presenting AF at the beginning of the procedure were electrically cardioverted and ablation was performed during sinus rhythm. Circumferential LA ablation lines were placed around the antrum of the ipsilateral pulmonary veins. An irrigated radiofrequency ablation catheter was used to deliver 25–35 W of power. Voltage and pace mapping along the ablation line were used to identify and close conduction gaps after circumferential line placement. The electrical isolation of all pulmonary veins with bidirectional block was verified with a multipolar circular mapping catheter and was defined as the procedural endpoint.

### LA size, function and strain assessed by 2D-STE

**Fig. 1:**
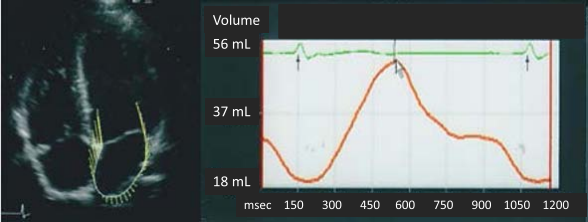
Representative image of time-left atrial volume curve. (Left) velocity vector imaging of the left atrium from the apical 4-chamber view; (Right) orange line: time-left atrial volume curve; green line: electrocardiogram. LA: left atrium

A standard echo-Doppler and two dimensional (2D)-STE examination were performed using a SC2000 ultrasound system (Siemens Medical Solutions Inc., Mountain View, CA, USA) with a 4V1c transducer (1.25–4.5 MHz). Echocardiographic measurements were made according to the American Society of Echocardiography criteria [19]. We examined the following parameters during sinus rhythm before PVI in apical 4-chamber view using offline software (Aquson, Sequoia, Siemens Medical Solutions Inc., Mountain View, CA, USA): maximum, pre-atrial contraction and minimum LAV; LA total and active emptying function (EF); longitudinal LA peak strain; and strain rate (SR) during systole and atrial contraction (Fig. 1). LAV, LA EF, longitudinal LA peak strain and SR were obtained from the time-LA volume curve and strain curve as we previously reported [2, 3, 16, 17]. LAV was indexed to body surface area. The reliability and the reproducibility of the STE method for quantification of LA function and volume were established in our previous study [2, 3, 16, 17]. Echocardiographic parameters were compared between the successful group (sinus rhythm was maintained for >1 year) and the recurrence group. The total and active LA EF were calculated as (maximum LAV – minimum LAV)/maximum LAV × 100 %, and (pre-atrial contraction LAV – minimum LAV)/pre-atrial contraction LAV × 100 %, respectively.

### Pulmonary capillary wedge pressure and LA stiffness estimated by 2D-STE

The combined assessment of LAV and LA function would be useful to estimate pre-atrial contraction LV filling pressure, because LAV and LA function are directly influenced by LV diastolic function. The KT index is calculated using the combination of LA function and volume. As LV diastolic function continues to decrease, LA volume continues to increase [20, 21]. Therefore, we employed LA volume as the denominator in the KT index to evaluate PCWP. Moreover, Hsiao et al. previously reported the logarithmic correlation between LV filling pressure and LA distensibility [(max LAVI – min LAVI)/min LAVI] that is similar to the total LAEF [(max LAVI –min LAVI)/max LAVI] [21]. Therefore, the KT index is defined as log_10_ (active LA EF/minimum LAV index) as we previously reported [16, 17]. Multivariate regression analysis showed that the ePCWP is calculated as 10.8 – 12.4 × KT index [16, 17]. The reliability of ePCWP has been well-established in several previous studies [16, 17, 22]. The ePCWP measured by this function just before right heart catheterization (within one hour) was strongly correlated with PCWP obtained by right heart catheterization (*r* = 0.92) in patients with normal sinus rhythm [16]. The intraobserver correlation coefficient and variability in ePCWP were 0.99 and 0.9 ± 1.5 %, respectively. The interobserver correlation coefficient and variability in ePCWP were 0.99 and 2.5 ± 1.8 %, respectively [16]. Moreover, we estimated LA stiffness using ePCWP as ePCWP/LA strain obtained by STE [23, 24].

### Statistical analyses

Continuous variables are expressed as the mean ± standard deviation, and categorical variables are presented as frequency and percentage. Differences in the categorical variables between the groups were tested by a Chi-square test or Fisher’s exact test, and differences in the continuous variables between the groups were tested by an unpaired *t*-test. The differences in CHA2DS2-VASc score between groups were tested by a Mann–Whitney *U*-test. A multivariate logistic regression analysis was performed using data from both groups to determine the independent echocardiographic predictors of successful AF ablation. Only parameters that were significant in univariate analysis were used in the multivariate regression model. Receiver operating characteristic (ROC) curve analysis was used to discriminate between the recurrence and successful groups using the various echo parameters, and we determined the sensitivity, specificity, positive and negative predictive values and area under the curve (AUC) for each parameter. Differences in AUC among the echo parameters were tested by the method established by Hanley and McNeil [25]. A p value <0.05 was considered significant. Statistical analyses were performed using Stat View version 5.0 (SAS Institution Inc., Cary, NC, USA).

## Results

Of 150 consecutive patients, 10 patients were excluded because of inadequate echo images of the LA (*n* = 6) and inability to perform echocardiography during sinus rhythm (*n* = 4). We excluded patients with severe mitral regurgitation (*n* = 2) and moderate or severe mitral stenosis (*n* = 1). Accordingly, we enrolled 137 patients (61 ± 10 years) who had echocardiography during sinus rhythm and underwent PVI for paroxysmal AF. Of 137 patients, 109 patients underwent their first AF ablation procedure and 28 patients underwent their second AF ablation procedure. Of 137 patients, 57 patients underwent right heart catheterization including PCWP measurement.

**Fig. 2:**
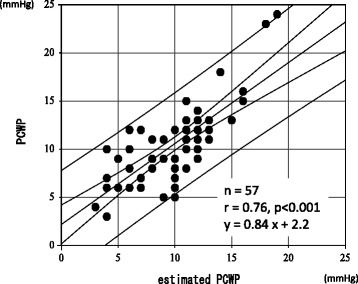
Relationship between pulmonary capillary wedge pressure measured by right heart catheterization and speckle tracking echocardiography. Center line indicates the regression line. Inner lines indicate the 95 % confidence intervals of the regression line. Outer lines indicate the 95 % confidence intervals of the raw data. PCWP: pulmonary capillary wedge pressure

The baseline characteristics of the study population are listed in Table 1. Echocardiographic parameters of patients with successful ablation and patients with AF recurrence are listed in Table 2. Thirty patients had AF recurrence (22 % of all patients) and the mean time from ablation to AF was 5 ± 2 months: all AF episodes were confirmed by ECG or Holter ECG. There was a strong correlation between PCWP obtained by right heart catheterization and ePCWP (*r* = 0.76, *p* < 0.001) in 57 patients (Fig. 2). The ePCWP, LA stiffness, maximum and minimum LAV index and the ratio of peak early transmitral flow velocity (E) to the regional tissue velocity of the mitral annulus measured during early filling (E/e’) before AF ablation in the recurrence group were increased compared with the successful group (Table 2). The total and active LA EF and longitudinal LA peak strain were decreased before AF ablation in the recurrence group. However, there was no significant difference in left ventricular ejection fraction or LA dimension between the two groups.

**Table 1:** Baseline characteristics of the study population

	Recurrence Group (*n* = 30)	Successful Group (*n* = 107)	*p* value
Age, years	59.4 ± 10.5	61.3 ± 10.7	0.22
Females, n (%)	9 (30)	31 (29)	0.91
Body mass index, kg/cm^2^	24.2 ± 3.3	23.9 ± 3.2	0.43
Heart rate, beat per minute	72 ± 15	73 ± 15	0.77
Systolic blood pressure, mmHg	121 ± 17	121 ± 15	0.83
Diastolic blood pressure, mmHg	75 ± 14	73 ± 11	0.45
eGFR, ml/min/1.73 m^2^	73.8 ± 16.2	72.8 ± 17.5	0.82
Previous ablation, n (%)	9 (30)	19 (18)	0.14
Hypertension, n (%)	14 (47)	49 (46)	0.93
Diabetes mellitus, n (%)	5 (17)	17 (16)	>0.99
Coronary artery disease, n (%)	2 (7)	6 (6)	>0.99
Smoking, n (%)	5 (17)	17 (16)	>0.99
Anti-arrhythmic drugs, n (%)	16 (53)	49 (46)	0.47
Congestive heart failure, n (%)	3 (10)	9 (8)	0.73
CHA2DS2-VASc score	1.4 ± 1.3	1.4 ± 1.2	0.83

*AF* atrial fibrillation, *eGFR* estimated glomerular filtration rate

**Table 2:** Comparison of echo parameters before AF ablation between in groups with successful and non-successful outcomes

	Recurrence Group (*n* = 30)	Successful Group (*n* = 107)	*p* value
LV EF, %	63 ± 8	64 ± 8	0.74
E/e’	12 ± 4	10 ± 3	0.039
LAD, mm	43 ± 5	41 ± 6	0.16
LV mass index, g/m^2^	97 ± 17	94 ± 17	0.41
Max LAVI, mL/m^2^	58 ± 15	49 ± 15	0.005
Min LAVI, mL/m^2^	37 ± 14	29 ± 12	0.002
Total LAEF, %	39 ± 13	44 ± 11	0.030
Active LAEF, %	20 ± 7	25 ± 10	0.033
Longitudinal LA peak strain	23 ± 9	28 ± 13	0.031
LA SR during systole, s^−1^	0.95 ± 0.42	1.12 ± 0.52	0.11
LA SR during AC, s^−1^	−0.84 ± 0.44	−1.01 ± 0.62	0.15
ePCWP, mmHg	14.6 ± 2.9	10.6 ± 3.5	<0.001
LA stiffness	0.83 ± 0.59	0.47 ± 0.33	<0.001

*AF* atrial fibrillation, *LVEF* left ventricular ejection fraction, *E*/*e*’ ratio of early filling velocity to myocardial tissue velocity, *LAD* left atrial dimension, *Max LAVI* maximum left atrial volume index, *Min LAVI* minimum left atrial volume index, *Total LAEF* left atrial total emptying fraction, *Active LAEF* left atrial active emptying function, *LA SR* left atrial strain rate, *AC* atrial contraction, *ePCWP* estimated pulmonary capillary wedge pressure

**Fig. 3:**
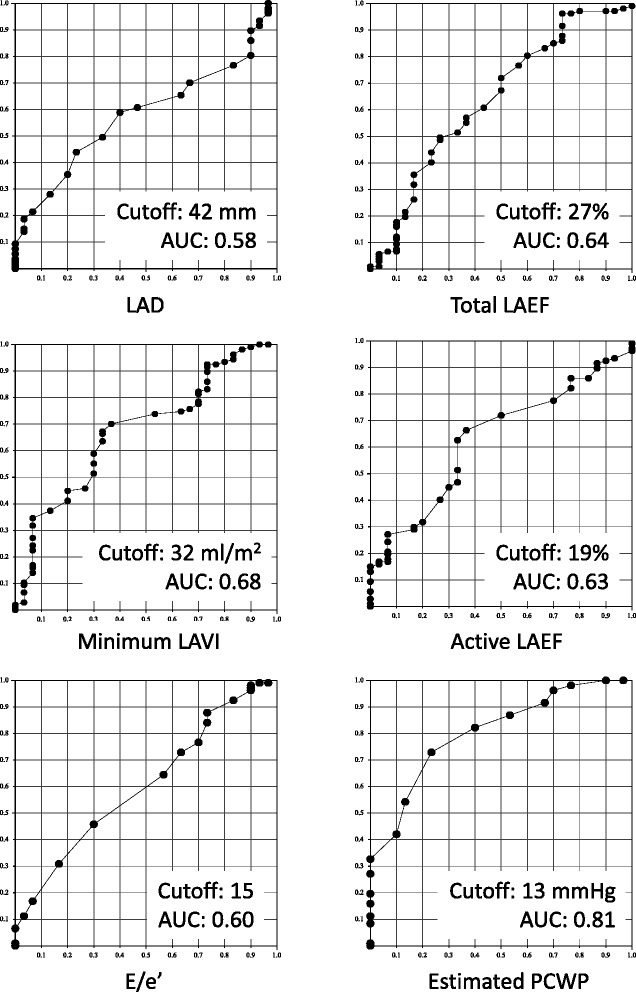
Receiver operating characteristic curve analyses of echo parameters for predicting successful AF ablation. LAD: left atrial dimension; AUC: area under the curve; Min LAVI: minimum left atrial volume index; Max LAVI: maximum left atrial volume index; LAEF: left atrial emptying fraction; PCWP: pulmonary capillary wedge pressure; E/e’: ratio of early filling velocity to myocardial tissue velocity

**Fig. 4:**
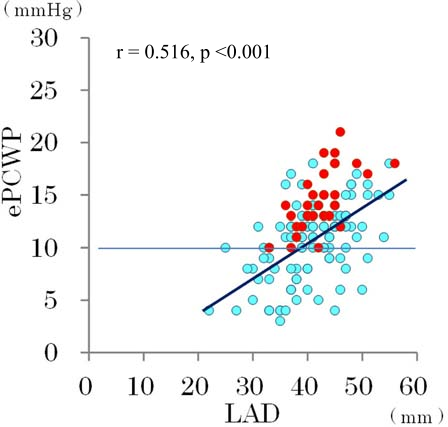
Left atrial diameter and pulmonary capillary wedge pressure as predictors of successful AF ablation. ePCWP: estimated pulmonary capillary wedge pressure; LAD: left atrial dimension; blue circle: AF recurrence; red circle: successful AF ablation; blue broken line shows 10 mmHg of ePCWP

Echocardiographic predictors of rhythm outcomes after AF ablation were evaluated by a multivariate logistic regression analysis (Table 3). All variables with a *p* value <0.05 in univariate testing were included in multivariate logistic regression analysis. However, maximum LAV index, active LAV index and total LA EF could not be included in the multivariate analysis due to multicollinearity. In multivariate analysis, ePCWP assessed during sinus rhythm before AF ablation had the most significant association with the outcome of AF ablation. Using 13 mmHg of ePCWP as the optimal cutoff value, the sensitivity and specificity for successful ablation were 73 and 77 %, respectively, and the positive and negative predictive values were 92 and 44 %, respectively. The AUC for ePCWP (0.81) was better than that for LAD (0.58, *p* < 0.001), total LA EF (0.64, *p* = 0.022), active LA EF (0.63, *p* = 0.010) and E/e’ (0.60, *p* = 0.044) (Fig. 3, Table 4). There was a tendency for the AUC for ePCWP to be better than that for minimum LAVI (0.68, *p* = 0.056). All patients with ePCWP <10 mmHg before AF ablation regardless of LA dimension had successful AF ablation (Fig. 4). Therefore, we also employed 10 mmHg as a second cutoff value for ePCWP to increase the positive predictive value rather than to maintain a high sensitivity. Using 10 mmHg of ePCWP as a second cutoff value, the sensitivity and specificity for successful ablation were 32 and 100 %, respectively, and the positive and negative predictive values were 100 and 29 %, respectively. Predictive accuracies of different ePCWP to predict successful AF ablation were shown in Table 5.

**Table 3:** Multivariate logistic regression analysis to determine the independent predictors of a successful AF ablation and subanalysis on first and second AF ablation

All patients (*n* = 137)				
	*p* value	Odds ratio	95 % confidence interval	
Minimum LAVI	0.011	1.093	1.021	1.171
ePCWP	<0.001	0.482	0.340	0.682
Longitudinal LA peak strain	0.072	0.940	0.879	1.005
E/e’	0.56	1.041	0.908	1.192
LA stiffness	0.19	0.296	0.047	1.851
First AF ablation (*n* = 109)				
	*p* value	Odds ratio	95 % confidence interval	
Minimum LAVI	0.029	1.092	1.009	1.182
ePCWP	<0.001	0.419	0.257	0.682
Longitudinal LA peak strain	0.094	0.930	0.855	1.012
E/e’	0.63	1.038	0.889	1.214
LA stiffness	0.39	0.377	0.041	3.462
Second AF ablation (*n* = 28)				
	*p* value	Odds ratio	95 % confidence interval	
Minimum LAVI	0.15	1.113	0.959	1.322
ePCWP	0.057	0.579	0.329	1.018
Longitudinal LA peak strain	0.97	0.967	0.845	1.107
E/e’	0.52	1.110	0.809	1.521
LA stiffness	0.30	0.122	0.002	6.697

*AF* atrial fibrillation, *ePCWP* estimated pulmonary capillary wedge pressure, *E*/*e*’ ratio of early filling velocity to myocardial tissue velocity, *LA* left atrial

**Table 4:** Sensitivity and specificity of echo parameters to predict successful AF ablation

	Cut off	Sensitivity %	Specificity %	PPV %	NPV %	AUC (95 % CI)
LAD, mm	42	60	59	29	84	0.58 (0.50 – 0.66)
Min LAVI, mL	32	67	66	36	88	0.68 (0.60 – 0.76)
Max LAVI, mL	51	70	60	33	88	0.66 (0.58 – 0.74)
Total LAEF, %	27	27	96	67	82	0.64 (0.56 – 0.72)
Active LAEF, %	19	63	66	35	87	0.63 (0.55 – 0.71)
ePCWP, mmHg	13	77	73	44	92	0.81 (0.74 – 0.88)

*AF* atrial fibrillation, *PPV* positive predictive value, *NPV* negative predictive value, *AUC* area under the curve, *LAD* left atrial dimension, *Min LAVI* minimum left atrial volume index, *Max LAVI* maximum left atrial volume index, *LAEF* left atrial emptying fraction, *ePCWP* estimated pulmonary capillary wedge pressure, *CI* confidence interval

**Table 5:** Predictive accuracies of different estimated pulmonary capillary wedge pressures to predict successful AF ablation

ePCWP	Sensitivity	Specificity	PPV	NPV
10 mmHg	32 (24 – 40)	100 (100 – 100)	100 (100 – 100)	29 (21 – 37)
11 mmHg	42 (34 – 50)	90 (85 – 95)	94 (90 – 98)	30 (22 – 38)
12 mmHg	54 (46 – 62)	87 (81 – 93)	93 (89 – 97)	34 (26 – 42)
13 mmHg	72 (64 – 80)	77 (70 – 84)	92 (87 – 97)	44 (36 – 52)
14 mmHg	82 (76 – 88)	60 (52 – 68)	88 (83 – 93)	49 (41 – 57)
15 mmHg	87 (81 – 92)	47 (39 – 55)	85 (79 – 91)	50 (42 – 58)
16 mmHg	92 (87 – 97)	34 (26 – 42)	83 (77 – 89)	53 (45 – 61)
17 mmHg	96 (93 – 99)	30 (22 – 38)	82 (76 – 88)	69 (61 – 77)
18 mmHg	98 (96 – 100)	24 (17 – 31)	80 (73 – 87)	77 (70 – 84)
19 mmHg	100 (100 – 100)	10 (5 – 15)	79 (72 – 86)	100 (100 – 100)

Data are percentages. Numbers in parentheses are 95 % confidence intervals. *AF* atrial fibrillation, *ePCWP* estimated pulmonary capillary wedge pressure, *PPV* positive predictive value, *NPV* negative predictive value

Note that the positive predictive values are useful to estimate the probability of successful AF ablation

## Discussion

The present study demonstrated that ePCWP estimated by the KT index was most useful predictor among echo parameters for successful AF ablation in patients with paroxysmal AF. The association between AF ablation outcome and ePCWP, calculated by the combination of LA function and volume, was stronger than the association observed using LA function and volume separately.

### AF ablation outcome and echocardiographic parameters

Recently, there were some studies that reported that LA function assessed by STE instead of LA size is a useful predictor for the outcome of AF ablation. Morris et al. reported that LA diastolic function assessed by LA strain (AUC: 0.827) and LA systolic function assessed by SR during atrial contraction (AUC: 0.806) in patients with paroxysmal AF and low CHADS2 score were useful predictors of AF ablation outcome [26]. Tops et al. reported that LA strain predicts reverse remodeling after AF ablation [27]. In the present study, LA strain was lower in the recurrence group than in the successful group, and this result is consistent with the findings of Morris et al. Furthermore, Montserrat et al. reported that patients with successful AF ablation had better total LA EF than those with AF recurrence, and total LA EF was an independent predictor of arrhythmia elimination after the first AF ablation [28]. Taken together, LA function is more associated with the outcome of AF ablation than LA size. However, regarding LA pressure, there are very few STE studies that evaluated noninvasive predictors of the successful AF ablation. The present study evaluated the predictive value of LA pressure on the success of AF ablation.

### AF ablation outcome and LA size and function

Among several echo parameters including estimated LA pressure and LA stiffness, we found that ePCWP is the most reliable echo parameter to predict AF ablation outcome. Using 13 mmHg of ePCWP as an optimal cutoff value, the positive predictive value for successful AF ablation was 92 % (95 % confidence interval: 87 – 97 %). Using 10 mmHg of ePCWP as a second optimal cutoff value, the positive predictive value for successful AF ablation was 100 %. These results suggest that ePCWP measured by STE before the procedure may be useful to predict of the outcome one year after AF ablation.

The ePCWP can be calculated by the KT index that consists of the combination of LA function (active LA EF) and LA volume (minimum LAV index). This suggests that LA pressure may be a more important factor for the outcome of AF ablation than LA function or volume evaluated separately. This also suggests that ePCWP may be the useful predictor of the outcome of AF ablation with high reliability, since the AUC was 0.81 based on ROC curve analysis. On the other hand, the other echo parameters had lower AUCs (0.58 – 0.68) than ePCWP.

In our previous study for the development of ePCWP, STE was performed just before right heart catheterization (within one hour). However, in the present study, STE was performed one day before AF ablation. Therefore, the correlation coefficient between ePCWP and PCWP measured by right heart catheterization was lower (*r* = 0.76) than the previous training study (*r* = 0.92) [16].

### Differences between E/e’ and ePCWP

E/e’ has been proposed to estimate LV filling pressure [29–31]. However, E/e’ does not reflect the condition of diastasis that may occur during mid and late diastole, or atrial contraction that is directly affected by LV stiffness. Therefore, E/e’ is a parameter that evaluates only early diastole. To overcome the limitations of E/e’, we proposed a combination of minimum LAVI and active LAEF (KT index) that evaluates LA features throughout diastole to estimate PCWP.

### AF ablation outcome and CHADS2 score

Kornej et al. reported that R2CHADS2 score, CHA2DS2-VASc score and LA dimension were significant predictors for late recurrence of AF after AF ablation. However, R2CHADS2 score and CHA2DS2-VASc score showed only low predictive value (AUC: 0.541 and 0.545, respectively) [13]. The statistical significance of the predictive value of these parameters despite a low AUC was due to a large population in the study [13].

### Study limitations

There are several limitations of this study. First, the study population was small, and only patients with paroxysmal AF were included. Therefore, there were no differences in clinical parameters between the two groups. Second, the rate of AF recurrence after AF ablation could have been underestimated. However, the protocol for detection of AF recurrence seems to be an ideal scenario from a practical point of view. Third, we focused only on echocardiographic parameters to predict the outcome of AF ablation. There is a possibility that other factors such as the duration of paroxysmal AF or the P wave duration may predict AF recurrence after ablation. Further study in a larger population including patients with persistent AF will be needed.

## Conclusions

An elevated ePCWP before AF ablation, but not an enlarged LA size, assessed by STE was the best predictor of AF recurrence after AF ablation, suggesting a strong relationship between LA pressure and the progression of LA remodeling responsible for AF. The echocardiographic parameter of ePCWP assessed by the KT index could be useful to improve candidate selection for AF ablation.

## Abbreviations

AF: atrial fibrillation
LA: left atrial
LAV: left atrial volume
PCWP: pulmonary capillary wedge pressure
ePCWP: estimated pulmonary capillary wedge pressure
KT index: kinetics-tracking index
STE: speckle tracking echocardiography
ECG: electrocardiogram
EF: emptying function
ROC: receiver operating characteristic
AUC: area under the curve
